# Intrasexual Vibrational Behavior of *Philaenus spumarius* in Semi-Field Conditions

**DOI:** 10.3390/insects12070584

**Published:** 2021-06-28

**Authors:** Imane Akassou, Sabina Avosani, Valentina Caorsi, Vincenzo Verrastro, Marco Ciolli, Valerio Mazzoni

**Affiliations:** 1 DICAM Department of Civil, Environmental and Mechanical Engineering, University of Trento, Via Mesiano 77, 38123 Trento, Italy; sabina.avosani@unitn.it (S.A.); marco.ciolli@unitn.it (M.C.); 2 Research and Innovation Centre, Fondazione Edmund Mach, Via Mach 1, 38098 San Michele all’Adige, Italy; valentina.zaffaronicaorsi@fmach.it (V.C.); valerio.mazzoni@fmach.it (V.M.); 3 CIHEAM—IAMB International Centre for Advanced Mediterranean Agronomic Studies, Via Ceglie 9, 70010 Bari, Italy; verrastro@iamb.it; 4 C3A, Centre Agriculture Food Environment, University of Trento, 38010 San Michele all’Adige, Italy

**Keywords:** vibrational signals, meadow spittlebug, intrasexual behavior, intrasexual interactions

## Abstract

**Simple Summary:**

Vibrational communication is widespread in insects. Since most studies of intrasexual behavior on insects have targeted few individuals, the behaviors occurring within groups have been left unexplored. Including multiple individuals in behavioral studies on vibrational communication would provide more reliable information about their intrasexual behavior. In semi-field conditions, the intrasexual behavior of the meadow spittlebug, *Philaenus spumarius*, was studied. Using a laser vibrometer, we recorded vibrational signals exchanged among individuals of the same sex, throughout their adult stage and during the day, from the morning to the evening. The results showed that males were less active than females and the interactions among males were less frequent than among females. Intrasexual interactions were characterized by signal overlapping in both unisex groups, in addition to signal alternating only in the case of males. This study provided a better understanding of the social behavior of *P. spumarius* based on vibrational signals.

**Abstract:**

Insects that communicate by vibrational signals live in a complex interactive network of communication. Most studies on insect intrasexual behavior, based on plant-borne vibrational signals, have targeted few individuals. Despite their importance, behaviors that occur within groups were often overlooked. The study of multiple individuals, when insects occur in high density could simulate the environment in which they live and provide more reliable information on their behavior. In semi-field conditions, we investigated the intrasexual behavior of the meadow spittlebug, *Philaenus spumarius.* Vibrational signals exchanged among individuals of the same sex were recorded throughout their adult stage, from late spring to early autumn, and during the day, from the morning to the evening using a laser vibrometer. Males were less active than females throughout the season and their interactions were less frequent compared to females. Intrasexual interactions were characterized by signal overlapping in both unisex groups, in addition to signal alternating only in the case of males. In conclusion, the study of signaling behavior in intrasexual groups contributed to a better understanding of *P. spumarius* social behavior. We discuss the hypothesis of a possible competitive behavior between males and cooperative behavior between females.

## 1. Introduction

Vibrational communication is an important and prevalent component in animal communication [1]. In nature, insects that communicate by vibrational signals live in a complex interactive environment, where they perceive signals from conspecifics, other species, and the surrounding environment [2,3]. Insects are therefore able to distinguish qualitative and quantitative characteristics of inter and intraspecific substrate-borne vibrations and react accordingly [4,5]. In fact, the exchange of vibrational signals occurs in different situations and can have several functions, such as attraction and localization of a mate [6,7], competition over a mate [7], coordination with conspecifics [8], and prey detection or predators avoidance [9].

Studying vibrational signals of insects in different contexts has provided relevant understanding of their behavior, ecology, and evolution. However, few studies have dealt with intrasexual interactions (e.g., [10]) and those that exist concern social and eusocial insects [11]. Studies of the vibrational behavior of insects, mostly focus on the mating behavior [12] and signals associated to pair formation, with bioassays that involve a pair (more often, a male and a female), sometimes in the presence of a third individual (e.g., [13,14]). Therefore, by targeting few individuals, behaviors that occur within groups are excluded even if for many species (especially of Hemipterans) the presence of groups on the same host plant is the rule and not the exception [15,16,17,18,19]. For this reason, the simulation of the environment in which insects live, by involving multiple individuals, could provide more reliable knowledge of their interactions that could be useful if applied to practical aspects that involve decision-making for conservation or pest control.

In this research, we aimed to investigate the vibrational behavior in a context of intrasexual groups in order to reveal the types of intrasexual signals and interactions. We chose, as a model species, the meadow spittlebug *Philaenus spumarius* (L.) (Hemiptera: Aphrophoridae). This insect is the major vector of *Xylella fastidiosa* subspecies *pauca*, the causal agent of the Olive Quick Decline Syndrome, a severe vascular disease that is leading to serious economic losses in olive production in Southern Italy [20,21] and poses severe risks to several other European countries [22]. The mating behavior of *P. spumarius* is mediated by vibrational signals and the role of intraspecific vibrational signals has already been described [23]. Since the calling behavior of females depends on their sexual maturation [24], their responsiveness to mating signals is delayed and increases through the season. Adults of *P. spumarius* can occur at high densities at the same and on nearby host plants [20,25,26,27,28], suggesting that their vibrational environment includes multiple individuals simultaneously signaling.

To study the vibrational signaling behavior of intrasexual groups of *P. spumarius*, we first investigated the signaling activity of males and females separately (in order to prevent mating) throughout the adult stage and during the day. Second, we characterized the types of signals emitted by both unisex groups and evaluated the effect of type of signals and number of signaling individuals on the number of emitted signals. Finally, we investigated the types of interactions that occurred among same-sex individuals signaling simultaneously. The adopted approach provided insights about the intrasexual behavior of *P. spumarius*, as it might be manifested in nature.

## 2. Materials and Methods

### 2.1. Insect Collection and Rearing

Second to fifth instar nymphs of *P. spumarius* were collected on their host plants from meadows in the Trentino region in Northeastern Italy from April to May 2018. Collected nymphs were transferred into mesh cages (Bugdorm−6620, 60 × 60 × 120 cm^3^, MegaView Science Co., Ltd., Xitun Dist., Taiwan) using a brush. Cages were supplied with *Vicia faba*, *Trifolium repens*, *Rumex spp* and *Helianthus annus* plants, and were maintained under controlled conditions (25 ± 2 °C, L16:D8, 75 ± 5% RH) in a glasshouse at Fondazione Edmund Mach (San Michele all’Adige, Trentino, Italy). After adult molting, insects were sexed and reared separately to prevent mating, according to conditions described in Avosani et al. [23].

### 2.2. Signal Recording

In each recording session, males (*n* = 10) and females (*n* = 10) were randomly collected from the rearing cages using a mouth aspirator and separately released into two mesh cages (Bugdorm−6620, 60 × 60 × 120 cm^3^, MegaView Science Co., Ltd., Xitun Dist., Taiwan), placed outdoor in a shaded area and always oriented in the same direction. Each cage contained a 1-year-old potted grapevine plant (*Vitis vinifera* L. cv. Pinot noir grafted on Kobber 5 BB) which was grown under greenhouse-controlled conditions (24 ± 1 °C, L16:D8, 75 ± 5% RH) and were not subjected to pesticide treatments. Cages were separated by a distance of 0.5 m in order to prevent any possible transmission of vibrations from one cage to another. At the end of each recording session, insects were put back in their rearing cages. Recordings were conducted outdoors to simulate field conditions.

Vibrational signals emitted by males and females were simultaneously recorded using two laser Doppler vibrometers (Ometron VQ−500-D-V Ltd., Coventry, UK, and PDV 100, Polytec, Inc., Dexter, MI, USA). Each laser was pointed at a reflective sticker attached to the stem (diameter 1 cm) of the grapevine plants. Since *P. spumarius* tended to move towards the green and tender apical shoots of the plants (personal observation), the sticker was placed approximately 10 cm below the apical shoot. Recordings were digitized with the software Pulse 21 (Brüel and Kjær Sound & Vibration A/S, Nærum, Denmark) at a 44.1 kHz sample rate and 16-bit depth resolution through a data acquisition device (LAN XI type 3050-B-040, Brüel and Kjær Sound & Vibration A/S, Nærum, Denmark), then stored onto a hard drive of a computer (HP, EliteBook 8460 p). To accommodate the processing of signals to manageable proportions, a “recording” was acquired as a 10 min segment.

### 2.3. Analyzed Parameters

To evaluate the insect signaling throughout the season, trials covered most of the insect adult stage, which was divided into two parts: “early” season (from 14 June to 31 July 2018), and “late” season (from 1 August to 28 September 2018). Early and late seasons corresponded to the periods associated with the absence and presence of ovarioles in females, respectively [24]. The signaling during nighttime was not evaluated, given that pilot recordings suggested that *P. spumarius* has a negligeable signaling activity during night (data not shown). The “recording session” consisted of three different periods of the day: morning (from 06:30 to 11:00), afternoon (from 11:30 to 16:00) and evening (from 16:30 to 21:00). Each recording session was replicated 12 times throughout the season, resulting in a total of 162 h for each sex (3 recording sessions × 4.5 h × 12 replicates).

In order to characterize the types of signals emitted by the tested males and females, using a random number generator, we randomly chose three recordings from each recording session, where insect vibrational signals occurred. In total, 74 recordings of females and 95 of males were used for data analysis. Analysis of signal spectrograms was performed with the software Raven Pro 1.4 (The Cornell Lab of Ornithology, 151 Ithaca, NY, USA) using Fast Fourier Transform type Hann, with 75% overlap and window length of 512 samples.

To investigate whether the co-presence of same-sex individuals on the same plant elicited signaling of other individuals and influenced the type of emitted signals, we assessed whenever possible the types of signals and number of signaling individuals. Vibrational signals were characterized according to Avosani et al., [23] as follows: female calling signal (FCS), female rejection signal (FRjS), male calling signal (MCS), male courtship signal (MCrS), and male-male signal (MMS). These signals can be composed of two elements, namely pulses (homogenous units of sound of specific duration [29]) and chirps (continuous sound characterized by a fundamental frequency and a clear harmonic structure [23]). To assess if signaling of two or more individuals tended to alternate or overlap, the type of interaction between signaling individuals was evaluated. In this regard, signals were ranked as “overlapped” when emitted at the same time by different individuals (the start and the end of the signals coincide by 0.05–0.1 s), “partially overlapped” when there was not a perfect overlap between the start and the end of the signals, and “alternated” when emitted with a delay of 0.5–1 s.

### 2.4. Statistical Analyses

Statistical analyses were performed with the software R version 4.0.2 (R Core Team, 2018) (R Foundation for Statistical Computing, Vienna, Austria) run in the R studio interface [30]. Plots and graphic design were done using R packages: “ggpubr”, “ggplot2” (R Foundation for Statistical Computing, Vienna, Austria) [31,32].

#### 2.4.1. Vibrational Signaling throughout the Season and during the Day

To explore the variation of signaling of both males and females, the signaling duration was calculated as the time that individuals spent signaling per recording session. The signaling duration of males and females was compared using the Wilcoxon signed-rank test. To evaluate the effect of date and period of the day on the signaling duration, we fitted a generalized least squares model for each sex using the function “gls” from the “nlme” package [32]. The signaling duration was used as the response variable, while the date was transformed into numbers and used as a numeric variable and the period of the day (morning, afternoon, and evening) was used as a categorical factor. Date for females and period for males were used as variance covariates. In the case of males, the model showed a significant effect of period, therefore we calculated pairwise comparisons among periods of the day using the R function “lsmeans” from the “lsmeans” package [33].

#### 2.4.2. Types of Signals throughout the Season

To compare the types of emitted signals in the season, the proportion of each type of signal per recording session was compared between “early” and “late” season using a Mann-Whitney two-sample test for each sex.

#### 2.4.3. Signaling According to the Number of Individuals and Type of Signals

To determine the effect of the number of signaling individuals and type of signals on the number of emitted signals, we conducted a Permanova test using the function “adonis” from the “vegan” package [34]. When the effect of a variable was significant, we applied a post hoc test: Mann-Whitney pairwise test, with Bonferroni correction. Whenever a variable did not meet the assumptions of the test, no further post hoc were applied.

#### 2.4.4. Types of Interactions

Finally, we performed a descriptive analysis of the type of interactions among signaling individuals for each sex (i.e., overlapped or alternated signals).

## 3. Results

### 3.1. Vibrational Signaling throughout the Season and during the Day

In our experiment, the signaling duration of males (mean ± SD: 20.51 ± 23.2 min) was significantly lower than that of females (mean ± SD: 60.17 ± 69.09 min) (Wilcoxon signed-rank test: W = 437, *p*-value = 0.005).

**Figure 1:**
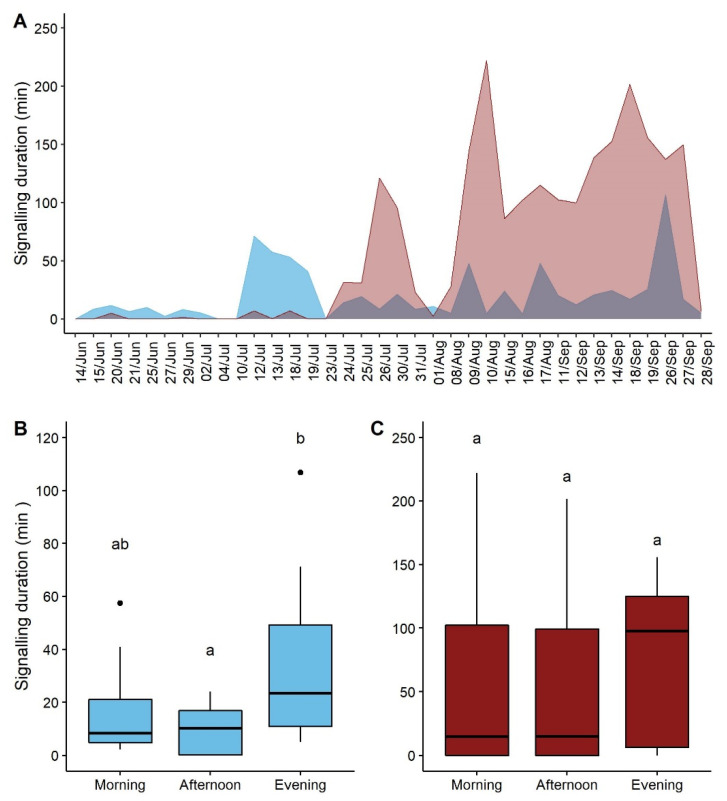
Vibrational signaling of males (blue) and females (red) of *P. spumarius* throughout the season and during the day: (**A**) Signaling duration per recording session of males and females; (**B**) Signaling duration of males per period of the day; (**C**) Signaling duration of females per period of the day indicate significant pairwise differences (*p* < 0.05). Black spots indicate outliers. Different letters (a, b) indicate significant pairwise differences (*p*-value < 0.05) between types of signals for each number of signaling individuals.

The signaling of both males and females differed within the season (F = 11.337, *p*-value = 0.002, F = 32.856, *p*-value < 0.001, males and females respectively) (Table 1: for model estimates). While males started to emit signals from the 15th of June, females rarely produced signals before the 24th of July 2018 (Figure 1A). The signaling of females significantly increased as the season progressed. A similar trend was observed in males although with much lower increasing rate (Figure 1A). The period of the day had a significant effect on the signaling duration of males (F = 4.728, *p*-value = 0.016) (Table 1, Table S1). The signaling duration in the evening was significantly longer than in the afternoon (*p*-value = 0.0342), while no differences in the males’ signaling duration were observed between morning and evening (*p*-value = 0.201) or morning and afternoon (*p*-value = 0.4412) (Figure 1C). The period of the day did not affect the females’ signaling duration (F = 0.243, *p*-value = 0.785) (Table 1, Table S1), even if the signaling activity tended to be higher in the evening, as shown by the median values (Figure 1B).

### 3.2. Types of Signals in “Early” and “Late” Season

**Figure 2:**
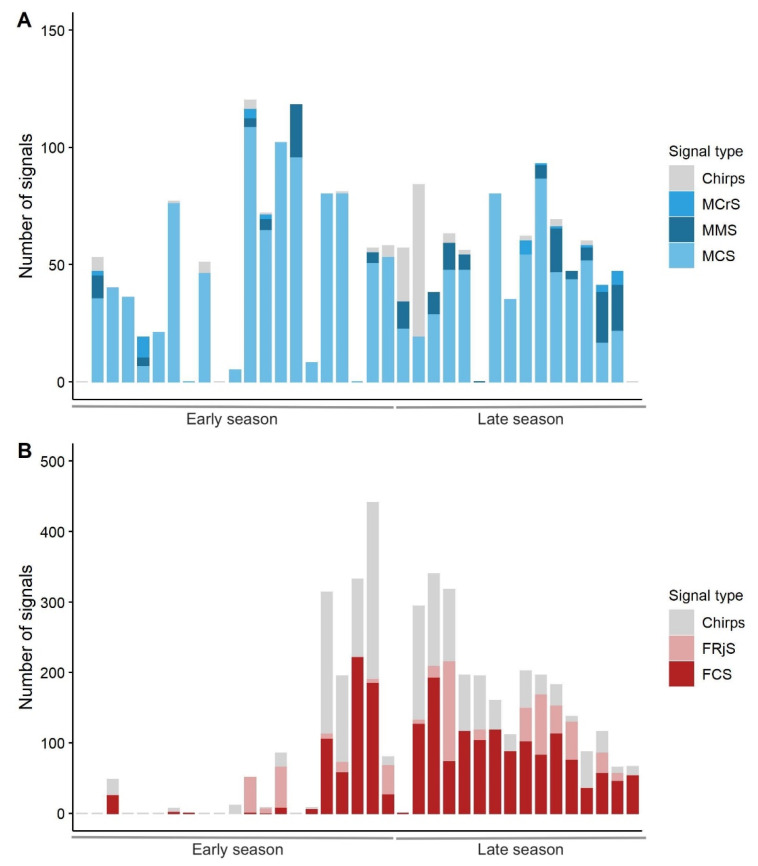
Number of signals per types emitted by males (**A**) and females (**B**) throughout the season. “Early” season: from 14th June to 31st of July 2018. “Late” season: from 1st of August to 28th of September 2018.

All types of previously described male signals (the MCS, the MMS and the MCrS [23]) were recorded during our trials. Nonetheless, males also emitted short sequences of chirps (2–3) or pulses (6–7) without a clear temporal pattern. The proportion of MMS was higher in the late season than in the early season (Mann-Whitney pairwise test, U = 10, *p*-value = 0.0312), while no significant differences in the number of chirps, MCS and MCrS were observed (Mann-Whitney pairwise test, U = 18, *p*-value = 0.281, U = 183.5, *p*-value = 0.066 and U = 15, *p*-value = 0.609, respectively) (Figure 2A).

All types of female signals were detected, except for FRsS (female response signal). Female signals (i.e., the FCS and the FRjS) are composed of chirps, of which repetition time and duration depend on the type of the signal [23]. In our trials, females produced sequences of 2–3 chirps, which differed from the FCS and the FRjS for their temporal features (time between chirps and duration of the signal sequence). The proportion of chirps was higher in the early season than in the late season (Mann-Whitney pairwise test, U = 116, *p*-value = 0.022), while the proportion of FCS was significantly lower in the early than in the late season (Mann-Whitney pairwise test, U = 32, *p*-value = 0.007). The proportion of emitted FRjS was similar between the early and late seasons (Mann-Whitney pairwise test, U = 43.5, *p*-value = 0.4347) (Figure 2B).

### 3.3. Signaling According to the Number of Individuals and Type of Signals

**Figure 3:**
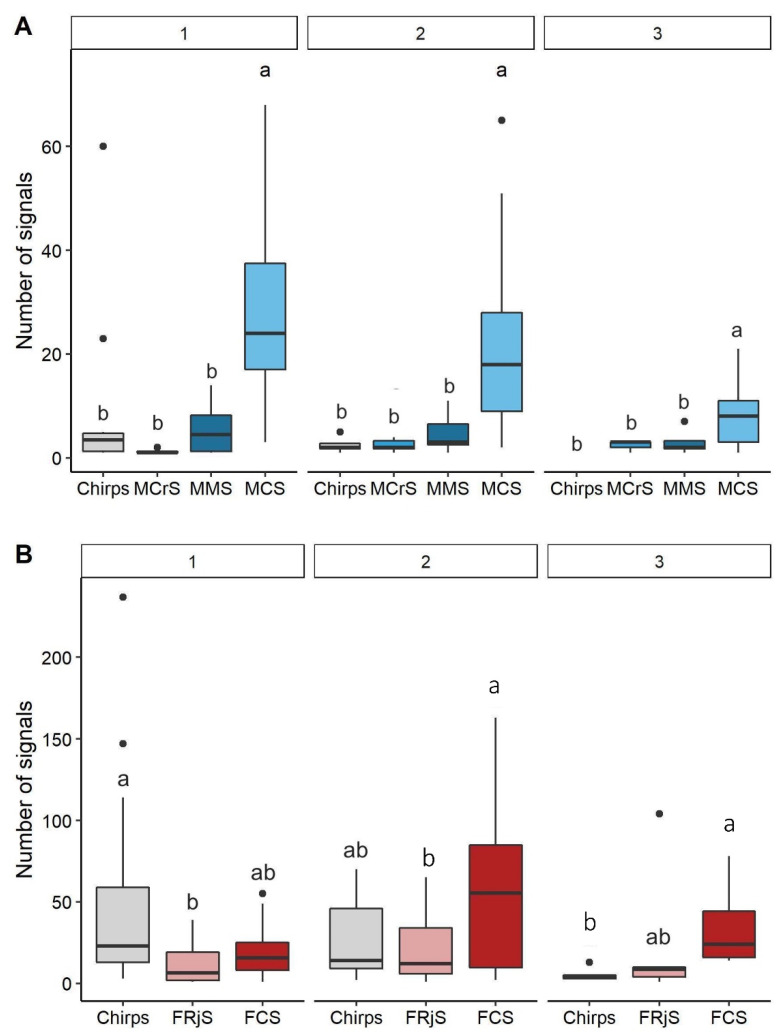
Number of signals emitted by males (**A**) and females (**B**) according to the type of signals and the number of signaling individuals (1, 2 and 3). Different letters (a, b) indicate significant pairwise differences (*p*-value < 0.05) between types of signals for each number of signaling individuals. Black spots indicate outliers.

The number of signals was significantly different between the type of signals emitted by males (Permanova test, F = 24.969, *p*-value = 0.001), as the number of MCS was higher than the number of MCrS (*p* < 0.001), MMS (*p*-value < 0.001) and isolated chirps (*p* < 0.001) (Figure 3A, see Table S2 for the other combinations). The effect of number of individuals on number of signals emitted was not considered (see Table S3 for complete results of the test), given the heterogeneity of dispersion found among the groups (permutation test for homogeneity of dispersion, F = 3.777, *p*-value = 0.023, Table S4). Furthermore, the interaction between type of signals and number of signaling individuals was not significant (F = 1.178, *p*-value = 0.287).

The number of signals emitted by females was significantly different between the type of signals (Permanova test, F = 3.161, *p*-value = 0.016) and the interaction between the type of signals and the number of signaling individuals was significant (Permanova test, F = 3.617, *p*-value = 0.001). The number of signaling individuals had no significant effect (Permanova test, F = 1.231, *p*-value = 0.280). Overall, females emitted fewer chirps than FCS and FRjS (Figure 3B). The type of signals depended on the number of individuals simultaneously signaling. In the case of one signaling female, the number of chirps was higher than the number of FRjS (*p*-value = 0.013), while no statistical difference was detected for the other combinations (*p*-value > 0.05). When two females were signaling, the number of signals was not statistically different among the types of signals (*p*-value > 0.05). When three individuals were signaling, females produced significantly more FCS than chirps (*p*-value = 0.005) while no statistical difference was detected among the other combinations (*p*-value > 0.05) (Figure 3B, Table S5).

### 3.4. Type of Interactions

Male interactions consisted mostly of overlapped signals (62.63%), but also on partially overlapped (20.62%) and alternated signals (16.75%) of same or different types. Females tended almost exclusively to overlap their signals (96.5%).

## 4. Discussion

This study investigated intrasexual communication and associated types of vibrational signals occurring in non-social insect groups. Under semi-field conditions (e.g., natural daylight and temperatures), the signaling behavior of *P. spumarius* females was recorded from the 24th of July 2018, earlier than a previous study in laboratory conditions that reported the occurrence of female mating signals from August, in correspondence with the maturation of their reproductive apparatus [24]. Females produced intersexual vibrational signals, when tested alone, in the presence of another male or when subjected to a playback only when they were sexually mature. Even if female signaling was mainly concentrated in the second part of the summer, their overall activity was significantly higher than that of males. Although the male signaling also increased during the summer, this trend was much stronger in females, who were basically silent during the first half of the season. The male signaling activity varied during the day, being higher in the evening and lower in the afternoon, while females tended to produce signals regardless of the time of the day. Unlike leafhoppers [7,35,36], *P. spumarius* females are the calling gender. The increased interest in finding a mate might explain why female signaling occurred during the entire day. Since *P. spumarius* can reach high population densities in the field [20], females likely dilute their signaling throughout the day to enhance their possibility to find a valuable partner, even if this strategy can be energetically costly [37]. Environmental conditions might have also affected the vibrational signaling of both males and females during the day and throughout the season, but their effect was not evaluated in this study.

Differently from Avosani et al. [23], where chirps were reported as short elements that compose the calling signals of both males and females of *P. spumarius*, chirps were detected in our trials also as isolated signals without a clear temporal pattern. Females produced fewer isolated chirps as the season progressed. Only in the later summer, when females had reached full sexual maturation, did they emit calling signals. Males, on the other hand, emitted isolated chirps without any seasonal trend, even if less frequently than females. Given that isolated chirps were produced by both sexes, the role of these signals remained unclear. One hypothesis is that chirps may be used to assess the presence of nearby conspecifics regardless of their sex. Although males and females could not directly interact, specific mating signals such as the female calling signal and the male courtship signal were recorded in our trials. As expected, the female response signal (which is emitted in tight synchrony within the male courtship to establish a duet [23]) was not produced by females. On the other hand, although the male courtship is usually emitted by males in response to female calling signals [23], it was produced by males in the absence of females during our trials. The same behavior was also observed in absence of other males and in response to playbacks of male signals [24]. It is possible that the male calling signal triggered other males to produce courtship signals, due to similarities with the female calling (i.e., they are both composed of chirps). However, a more probable hypothesis is that the courtship signal (and with some degree the female calling signal as well), could be used by the spittlebugs to assess the behavior of their (same sex) neighbors. In fact, insects may adjust their signals to the same level (cooperative interaction) or modify the spectral and temporal features of their signal to decrease their rival fitness (competitive interaction) [38,39]. In some insect species, collective signaling behaviors allow them to regulate local population densities and assess competitiveness of neighbors over food, mate or space [40,41]. When two individuals approach one another or come in contact, an aggressive behavior can also be manifested [40]. In our study, *P. spumarius* females and males expressed aggression by emitting female rejection signals and male-male signal, respectively.

Males of *P. spumarius* produced, overall, more male calling signals, independently from the number of signaling individuals. Contrary to our expectations, the male signaling activity was lower when more individuals were simultaneously signaling, suggesting that they may avoid interacting with their signaling neighbors. The situation was different in the case of females. When females emitted signals alone, they produced more chirps, while they emitted more female calling signals when interacting with other females. The presence of signaling females may trigger the activity of others, creating a cooperation that may increase their chances of finding/attracting a suitable mate [40]. Moreover, the cooperation expressed as a possible signaling chorus would enlarge the female active space (the three dimensional area over which a signal can be detected by a potential receiver) on the same plant, overcoming the energetic cost of signaling throughout the day [42,43]. Playback bioassays of the different types of signals, using same-sex groups, would confirm or reject these hypotheses.

Aside from the type of emitted signals, our study demonstrated that signals emitted by individuals of the same sex during the same time window were alternated or overlapping. Alternated signals in the case of males may impair the signaling of others [44], particularly, as mentioned before, that male activity was reduced when more individuals were signaling. Furthermore, alternated signals may also refer to competition between males over space on the same plant. When males are clustered in space, they strongly compete over their territory [39]. On the other hand, the overlap of signals that occurred in both intrasexual groups may underlie a cooperative behavior driving adults of *P. spumarius* to enhance their feeding by aggregation on the same part of the plant. *Philaenus spumarius* is a xylem-feeder that requires a great amount of energy to overcome the high tension in the xylem mainstream of the plant [45]. The potential aggregation of adults on the same plant might be a strategy to overcome the xylem tension that results in less energy expenditure [20]. A similar strategy is used by the group-living treehopper *Calloconophora pinguis*, in which nymphs overlap their vibrational signaling to recruit further members to a new feeding source [8]. Moreover, this behavior may also increase the fitness of the spittlebug by protecting them from predators. Nymphs of *P. spumarius* aggregate on the same plant and share the same spittle mass, which provides them with a shelter and protects them from natural enemies [46,47]. Similarly, adults could prevent being localized by natural enemies by synchronizing their signals [42].

## 5. Conclusions

Investigating the intrasexual behavior of *P. spumarius*, based on vibrational signals, allowed us to discern substantial behavioral differences between males and females and revealed that the type of intrasexual interactions that occur among simultaneously signaling males consisted of overlapping and alternated signals, while they consisted of only overlapping signals in the case of females. Besides providing ethological insights, similar information may support the development of behavioral manipulation techniques based on vibrations. By exploiting the competitive or cooperative behavior to target individuals of the same sex [48]. Further research based on vibrational signal playbacks is needed to determine the exact function of these intrasexual interactions in the case of *P. spumarius*.

## Figures and Tables

**Table 1:** Estimated regression coefficients, standard error, z ratio and *p*-value from the gls model testing the effect of date and period of the day on the number of signals. The intercept corresponds to the period afternoon.

Sex	Parameter	Estimate	Std. Error	T-Value	*p*-Value
Males	Intercept	1.087	3.174	0.342	0.734
	Date	0.174	0.052	3.368	0.001
	Period Evening	24.967	8.656	2.884	0.007
	Period Morning	7.078	5.626	1.258	0.217
Females	Intercept	−1.271	1.099	−1.157	0.256
	Date	1.041	0.191	5.427	<0.001
	Period Evening	−2.010	6.349	−0.316	0.753
	Period Morning	−1.522	2.305	−0.660	0.514

## Data Availability

The data that support the findings of this study are available from the corresponding author, upon request and agreement of the authors.

## Supplementary Materials

The following are available online at https://www.mdpi.com/article/10.3390/insects12070584/s1, Table S1: Signaling activity per period of the day as mean ± standard deviation (SD), Table S2: Complete results of the Mann-Whitney pairwise test, with Bonferroni correction, to test differences in the type of signals for both sexes, Table S3: Complete results of the Permanova test of the number of signals for both sexes, Table S4: Complete results of the analysis of multivariate homogeneity of group dispersions for both sexes as an assumption for the Permanova test. In the case of males, the heterogeneity of dispersion was significant for the number of individuals. Therefore, the low *p*-value in the Permanova test was not considered and no further pairwise test was conducted for this variable, Table S5: Complete results of the Mann-Whitney pairwise tests, with Bonferroni correction, to test differences in the type of signals in each number of signaling individuals in the case of females.

## References

[1] Hill P.S. (2008). Vibrational Communication in Animals.

[2] Čokl A., Virant-Doberlet M. (2003). Communication with substrate-borne signals in small plant-dwelling insects. Annu. Rev. Entomol..

[3] Virant-Doberlet M., Mazzoni V., De Groot M., Polajnar J., Lucchi A., Symondson W.O., Čokl A., Cocroft R.B., Gogala M., Hill P.S.M., Wessel A. (2014). Vibrational communication networks: Eavesdropping and biotic noise. Studying Vibrational Communication.

[4] Castellanos I., Barbosa P. (2006). Evaluation of predation risk by a caterpillar using substrate-borne vibrations. Anim. Behav..

[5] Evans T.A., Inta R., Lai J.C., Prueger S., Foo N.W., Fu E.W.e., Lenz M. (2009). Termites eavesdrop to avoid competitors. Proc. R. Soc. B Biol. Sci..

[6] Mazzoni V., Lucchi A., Ioriatti C., Virant-Doberlet M., Anfora G. (2010). Mating behavior of *Hyalesthes obsoletus* (Hemiptera: Cixiidae). Ann. Entomol. Soc. Am..

[7] Mazzoni V., Presern J., Lucchi A., Virant-Doberlet M. (2009). Reproductive strategy of the nearctic leafhopper *Scaphoideus titanus* Ball (Hemiptera: Cicadellidae). Bull. Entomol. Res..

[8] Cocroft R.B. (2005). Vibrational communication facilitates cooperative foraging in a phloem-feeding insect. Proc. R. Soc. B Biol. Sci..

[9] Virant-Doberlet M., Kuhelj A., Polajnar J., Šturm R. (2019). Predator-prey interactions and eavesdropping in vibrational communication networks. Front. Ecol. Evol..

[10] Bedoya C.L., Brockerhoff E.G., Hayes M., Leskey T.C., Morrison W.R., Rice K.B., Nelson X.J. (2020). Brown marmorated stink bug overwintering aggregations are not regulated through vibrational signals during autumn dispersal. R. Soc. Open Sci..

[11] Hill P.S.M., Mazzoni V., Narins P., Virant-Doberlet M., Wessel A., Hill P.S.M., Lakes-Harlan R., Mazzoni V., Narins P.M., Virant-Doberlet M., Wessel A. (2019). Quo Vadis, Biotremology?. Biotremology: Studying Vibrational Behavior.

[12] Virant-Doberlet M., Cokl A. (2004). Vibrational communication in insects. Neotrop. Entomol..

[13] Mazzoni V., Lucchi A., Čokl A., Prešern J., Virant-Doberlet M. (2009). Disruption of the reproductive behaviour of *Scaphoideus titanus* by playback of vibrational signals. Entomol. Exp. Appl..

[14] Kuhelj A., Virant-Doberlet M. (2017). Male–male interactions and male mating success in the leafhopper *Aphrodes makarovi*. Ethology.

[15] Addesso K.M., McAuslane H.J., Cherry R. (2012). Aggregation behavior of the southern chinch bug (Hemiptera: Blissidae). Environ. Entomol..

[16] Biedermann R. (2003). Aggregation and survival of *Neophilaenus albipennis* (Hemiptera: Cercopidae) spittlebug nymphs. Eur. J. Entomol..

[17] Kusmayadi A., Kuno E., Sawada H. (1990). The spatial distribution pattern of the brown planthopper *Nilaparvata lugens* Stål (Homoptera: Delphacidae) in west Java, Indonesia. Popul. Ecol..

[18] Park Y.-L., Perring T.M., Farrar C.A., Gispert C. (2006). Spatial and temporal distributions of two sympatric *Homalodisca* spp.(Hemiptera: Cicadellidae): Implications for areawide pest management. Agric. Ecosyst. Environ..

[19] Pérez-Rodríguez J., Martínez-Blay V., Soto A., Selfa J., Monzó C., Urbaneja A., Tena A. (2017). Aggregation patterns, sampling plan, and economic injury levels for the new citrus pest *Delottococcus aberiae* (Hemiptera: Pseudococcidae). J. Econ. Entomol..

[20] Cornara D., Bosco D., Fereres A. (2018). *Philaenus spumarius*: When an old acquaintance becomes a new threat to European agriculture. J. Pest Sci..

[21] Saponari M., Loconsole G., Cornara D., Yokomi R.K., De Stradis A., Boscia D., Bosco D., Martelli G.P., Krugner R., Porcelli F. (2014). Infectivity and transmission of *Xylella fastidiosa* by *Philaenus spumarius* (Hemiptera: Aphrophoridae) in Apulia, Italy. J. Econ. Entomol..

[22] Schneider K., Van der Werf W., Cendoya M., Mourits M., Navas-Cortés J.A., Vicent A., Oude Lansink A. (2020). Impact of *Xylella fastidiosa* subspecies *pauca* in European olives. Proc. Natl. Acad. Sci. USA.

[23] Avosani S., Daher E., Franceschi P., Ciolli M., Verrastro V., Mazzoni V. (2020). Vibrational communication and mating behavior of the meadow spittlebug *Philaenus spumarius*. Entomol. Gen..

[24] Avosani S., Franceschi P., Ciolli M., Verrastro V., Mazzoni V. (2021). Vibrational playbacks and microscopy to study the signalling behaviour and female physiology of *Philaenus spumarius*. J. Appl. Entomol..

[25] Bodino N., Cavalieri V., Dongiovanni C., Plazio E., Saladini M.A., Volani S., Simonetto A., Fumarola G., Di Carolo M., Porcelli F. (2019). Phenology, seasonal abundance and stage-structure of spittlebug (Hemiptera: Aphrophoridae) populations in olive groves in Italy. Sci. Rep..

[26] Bodino N., Cavalieri V., Dongiovanni C., Saladini M.A., Simonetto A., Volani S., Plazio E., Altamura G., Tauro D., Gilioli G. (2020). Spittlebugs of Mediterranean olive groves: Host-plant exploitation throughout the year. Insects.

[27] Mangan R., Wutz A. (1983). Aggregation patterns of meadow spittlebugs, *Philaenus spumarius* L. (Homoptera: Cercopidae), on old-field alfalfa plants. Environ. Entomol..

[28] Weaver C.R., King D. (1954). Meadow spittlebug, *Philaenus leucophthalmus* (L.). Ohio Agric. Exp. Stn. Res. Bull..

[29] Broughton W. (1963). Method in bio-acoustic terminology. Acoust. Behav. Anim..

[30] Team R.C. (2020). R: A Language and Environment for Statistical Computing.

[31] Wickham H. (2011). ggplot2. Wiley Interdisciplinary Reviews. Comput. Stat..

[32] Pinheiro J., Bates D., DebRoy S., Sarkar D., Team R.C. (2007). Linear and nonlinear mixed effects models. R Package Version.

[33] Lenth R., Lenth M.R. (2018). Package ‘lsmeans’. Am. Stat..

[34] Oksanen J., Blanchet F.G., Kindt R., Legendre P., Minchin P.R., O’hara R., Simpson G.L., Solymos P., Stevens M.H.H., Wagner H. (2013). Package ‘vegan’. Community Ecol. Package Version.

[35] Legendre F., Marting P.R., Cocroft R.B. (2012). Competitive masking of vibrational signals during mate searching in a treehopper. Anim. Behav..

[36] Nieri R., Mazzoni V., Gordon S.D., Krugner R. (2017). Mating behavior and vibrational mimicry in the glassy-winged sharpshooter, *Homalodisca vitripennis*. J. Pest Sci..

[37] Kuhelj A., De Groot M., Pajk F., Simčič T., Virant-Doberlet M. (2015). Energetic cost of vibrational signalling in a leafhopper. Behav. Ecol. Sociobiol..

[38] Greenfield M.D. (1994). Cooperation and conflict in the evolution of signal interactions. Annu. Rev. Ecol. Syst..

[39] West-Eberhard M.J. (1984). Sexual selection, competitive communication and species specific signals in insects. Insect Communication, Proceedings of the 12th Symposium of the Royal Entomological Society of London, London, UK, 7–9 September 1984.

[40] Greenfield M.D. (2002). Signalers and Receivers: Mechanisms and Evolution of Arthropod Communication.

[41] Wynne-Edwards V.C. (1962). Animal Dispersion: In Relation to Social Behaviour.

[42] Greenfield M.D. (1994). Synchronous and alternating choruses in insects and anurans: Common mechanisms and diverse functions. Am. Zool..

[43] Mazzoni V., Eriksson A., Anfora G., Lucchi A., Virant-Doberlet M. (2014). Active space and the role of amplitude in plant-borne vibrational communication. Studying Vibrational Communication.

[44] Hunt R.E., Morton T.L. (2001). Regulation of chorusing in the vibrational communication system of the leafhopper *Graminella nigrifrons*. Am. Zool..

[45] Malone M., Watson R., Pritchard J. (1999). The spittlebug *Philaenus spumarius* feeds from mature xylem at the full hydraulic tension of the transpiration stream. New Phytol..

[46] Wise M.J., Kieffer D.L., Abrahamson W.G. (2006). Costs and benefits of gregarious feeding in the meadow spittlebug, *Philaenus spumarius*. Ecol. Entomol..

[47] McEvoy P.B. (1986). Niche partitioning in spittlebugs (Homoptera: Cercopidae) sharing shelters on host plants. Ecology.

[48] Nieri R., Anfora G., Mazzoni V., Rossi Stacconi M.V. (2021). Semiochemicals, semiophysicals and their integration for the development of innovative multi-modal systems for pest monitoring and control. Entomol. Gen..

