# Supplementary material for: Intrasexual Vibrational Behavior of Philaenus spumarius in Semi-Field Conditions
Source: Insects. 2021 Jun 28;12(7):584. doi: 10.3390/insects12070584 (PMC8306748; doi:10.3390/insects12070584)
Supplement: Supplementary file 1 [file insects-12-00584-s001.zip › insects-1228972-supplementary.pdf]

Supplementary Material: Intrasexual vibrational behavior of *Philaenus spumarius* in semi-field conditions

Imane Akassou, Sabina Avosani, Valentina Caorsi, Vincenzo Verrastro, Marco Ciolli, and Valerio Mazzoni

Table S1: Signaling activity per period of the day as Mean ± standard deviation (SD).

Sex	Period of the day	Mean ± SD	n
Males	Morning	16.75±17.10	12
	Afternoon	9.86±8.75	12
	Evening	34.93±31.27	12
Females	Morning	53.32±74.41	12
	Afternoon	53.12±72.33	12
	Evening	74.07±64.04	12

Table S2. Complete results of the Mann–Whitney pairwise test, with Bonferroni correction, to test differences in the type of signals for both sexes.

Sex	Contrast	*p*-value
Males	MCrS- Chirps	1.00
	MCS- Chirps	2.0e-05
	MMS - Chirps	1.00
	MCS- MCrS	4.1e-07
	MMS- MCrS	0.11
	MMS- MCS	1.1e-08
Females	FCS- Chirps	1.0000
	FRjS- Chirps	0.0734
	FRjS- FCS	0.0065

Table S3. Complete results of the Permanova test of the number of signals for both sexes. Bold numbers indicate significant differences.

Sex	Parameter	Df	SumsOfSqs	MeanSqs	F.Model	R2	Pr(>F)
Males	Signal	3	8.5082	2.83608	24.9689	0.34154	0.001
	Individuals	2	1.1953	0.59765	5.2617	0.04798	0.002
	Signal: Individuals	5	0.6692	0.13385	1.1784	0.02686	0.287
	Residuals	128	14.5388	0.11358		0.58362	
	Total	138	24.9116			1.00000	
Females	Signal	2	1.0190	0.50952	3.1607	0.04152	0.016
	Individuals	2	0.3971	0.19854	1.2316	0.01618	0.280
	Signal:Individuals	4	2.3326	0.58314	3.6174	0.09504	0.001
	Residuals	129	20.7954	0.16120		0.84727	
	Total	137	24.5441			1.00000	

Table S4. Complete results of the analysis of multivariate homogeneity of group dispersions for both sexes as an assumption for the Permanova test. In the case of males the heterogeneity of dispersion was significant for the

number of individuals. Therefore, the low *p*-value in the Permanova test was not considered and no further pairwise test was conducted for this factor. Bold number indicate significant differences.

Sex	Parameter		Df	Sum Sq	Mean Sq	F	N.Perm	Pr(>F)
Males	Type of signals	Groups	3	0.0831	0.028	0.7256	999	0.569
		Residuals	135	5.1520	0.038			
	Number of individuals	Groups	2	0.13423	0.067	3.7768	999	**0.023**
		Residuals	136	2.41668	0.018			
Females	Type of signals	Groups	2	0.0381	0.019	0.5427	999	0.577
		Residuals	135	4.7407	0.035			
	Number of individuals	Groups	2	0.0438	0.022	0.5472	999	0.566
		Residuals	135	5.4002	0.0402			

Table S5. Complete results of the Mann-Whitney pairwise test, with Bonferroni correction, to test differences in the type of signals in each number of signaling individuals in the case of females.

Number of individuals	Contrast	*p*-value
1	FCS- Chirps	0.172
	FRjS- Chirps	0.013
	FRjS- FCS	0.278
2	FCS- Chirps	0.119
	FRjS- Chirps	1.000
	FRjS- FCS	0.072
3	FCS- Chirps	0.006
	FRjS- Chirps	1.000
	FRjS- FCS	0.082
